# Older persons making a life closure: experiences of loneliness in an academic nursing home – a phenomenological qualitative interview study

**DOI:** 10.1080/17482631.2024.2398201

**Published:** 2024-09-01

**Authors:** Annika Söderman, Mialinn Arvidsson-Lindvall

**Affiliations:** a Faculty of Medicine and Health, School of Health Sciences, Örebro University, Örebro, Sweden; b Faculty of Medicine and Health, University Health Care Research Centre, Örebro, Sweden; c School of Health, Care and Social Welfare, Mälardalen University, Västerås, Sweden

**Keywords:** Experiences, loneliness, municipality health care, nursing homes, older persons

## Abstract

**Background:**

Loneliness is a public health concern and more than half of the residents in nursing homes experience lonliness. Risk factors are age and loss of close relatives.

**Purpose:**

This study aimed to describe experiences of loneliness among older people living in an academic nursing home.

**Methods:**

Qualitative semi-structured interviews were conducted with ten older people and data analysed with systematic text condensation inspired by a phenomenological approach.

**Results:**

Three themes were identified: “Relatives and health care professionals matter”; “Acceptance and meaningful existence alleviate loneliness”; and “Challenges affecting the experience of loneliness”. The older persons described themselves as lonely, but their experience of loneliness differed. They managed loneliness by adapting to it or getting used to it; some also chose to be alone. To add meaningfulness to their daily life, talking about memories and their past were appriciated. Personality traits and variations in functional ability were identified as barriers to social interactions.

**Conclusions:**

Health care professionals can reduce negative experiences of loneliness by listening to nursing home residents, creating a meaningful daily life with individualized activities, and by encouraging contacts with close relatives. This can be a way of maintaining older persons’ dignity and coping with the longing for what has been.

## Background

Loneliness is generally understood as the discrepancy between a person’s preferred and their actual level of social contact (Peplau & Perlman, 1982). Even in older persons who have daily activities and are included in a social context with other people around, a feeling of loneliness can emerge (Larsson et al., 2023). Older persons are vulnerable through loneliness, as loneliness can cause great suffering in the individual (Sundström et al., 2018). To address older persons’ feelings of loneliness and investigate when and how they experience aspects of loneliness, the feeling of loneliness needs to be investigated and described from the perspective of the older persons themselves. With this perspective a deeper understanding can be created of what help they would need.

### Prevalence of and risk factors for loneliness

Age is the greatest risk factor for loneliness. A high prevalence of loneliness is found in individuals in their 20s; this starts to decline in middle age but rises again around the age of 80, when it reaches the highest levels (Luhmann & Hawkley, 2016; Shovestulm et al., 2020). Approximately 29% of people older than 60 years and living in their own home feel lonely, 7.9% of whom have been reported to have severe feelings of loneliness in an international comparison (Chawla et al., 2021). Loneliness is therefore a public health problem and chronic loneliness in people over 60 years leads to more physician visits, but is not associated with more hospital admissions (Gerst-Emerson & Jayawardhana, 2015).

During 1992–2014 the prevalence of loneliness (feeling lonely often or always) for people older than 77 years in Sweden was 8–11%. There is a clear connection between loneliness and the loss of a close relative (Dahlberg et al., 2018). Hawkley et al. (2019) show stable prevalence over time and report that loneliness is associated with low income, living alone and having few social contacts. Loneliness is also a risk factor for the transition into frailty in older people (Sha et al., 2020). Older people with recurring and extensive health and care needs are referred to as “the sickest older persons” or “frail older persons” (Social Health and Welfare, 2011). Older persons’ experiences of loneliness also differ depending on the health care context they are in (Larsson et al., 2023).

### Loneliness in nursing homes

Feeling lonely can be experienced differently and loneliness is one of the most prevalent problems encountered by older individuals in nursing homes (Huang et al., 2022; Mansfield et al., 2021; Zhang et al., 2023). Some people feel lonely when alone, while others may feel lonely when surrounded by other people. For older persons living in nursing homes, assisted living and other long-term care environments, feelings of loneliness can be exacerbated when actual social relations fall short of what is desired (Cacioppo et al., 2015). People living in nursing homes have been reported to experience loneliness twice as often as do people living at home, and to have a feeling of confinement, while people living at home experience loneliness based on ageing, discrimination and avoidance (Shpakou et al., 2021). Loneliness in nursing homes can be linked to depression, suicidal thoughts and frailty (Lapane et al., 2022). On the other hand, loneliness can be a reason to move into a nursing home (Eskimez et al., 2019), while half of those who already live there are bothered by anxiety and loneliness (Social Health and Welfare, 2020b). Internationally, on average 61% of nursing home residents are moderately and 35% severely lonely (Gardiner et al., 2020). In Sweden, approximately 108 000 persons live in nursing homes (Social Health and Welfare, 2020b). Altogether, the context can have impact on feelings of loneliness and earlier research suggest that by identifying the contextual components associated with a reduction in loneliness is important (Hoang et al., 2022).

### Different dimensions of loneliness

Loneliness has been described as a subjective and existential feeling, a feeling of being alone where the person does not get their social needs satisfied, which can lead to anxiety (Perlman & Peplau, 1981). Loneliness differs from social isolation, and being alone, which is objectively measured with outcomes such as the number of social contacts and which is a more neutral phenomenon, as it can be perceived as both positive and negative (Bolmsjö et al., 2019). The concepts are interconnected as social isolation is a risk factor for loneliness (Machielse, 2015). Existential loneliness is defined as “an immediate awareness of being fundamentally separate from other people and from the universe” (Bolmsjö et al., 2019, p. 1322). This awareness usually gives rise to negative moods and emotions (Bolmsjö et al., 2019).

Loneliness is a public health problem as a large proportion of older persons experience loneliness. Even though loneliness is more common among persons in nursing homes, previous research on loneliness is mostly based on persons who live in their own homes (Shpakou et al., 2021). The research also largely focuses on prevalence and causes. For increased knowledge and understanding of loneliness, there is a need to further study loneliness in the daily life of older people living in nursing homes. More knowledge can enable both health care and society to work preventively and alleviate loneliness to avoid suffering in older persons and help them maintain their quality of life. Therefore, the aim of this study was to describe the experience of loneliness in older persons living in a nursing home. The main objectives were: Do people living in an academic nursing home experience loneliness? How do people describe their experience of loneliness?

## Methods

### Design

A qualitative descriptive interview study was performed using thematic analysis with systematic text condensation inspired by a phenomenological approach (Malterud, 2012), examining older persons’ lived experiences, and creating an understanding of these experiences.

### Context

The management of an academic nursing home initiated the study as they were interested in exploring loneliness in older persons. In Sweden, the term “academic nursing home” refers to a nursing home where there is cooperation between the municipality, the university and in some cases the region to facilitate for research, development, and education, to create quality in the care for older people. Compared to other ordinary nursing homes, this form of collaboration supports the academic nursing home to develop and to work actively with research. The cooperation, in the specific region for this study, was led by a forum where managers and/or researchers from all three areas were involved. The managers of the academic nursing home contacted researchers in the forum to achieve help to conduct the study.

The setting was a nursing home in a medium-sized city in central Sweden. The nursing home has 81 apartments (including bedroom with bathroom and kitchenette) with room for 85 older persons. The apartments were incorporated in one nursing home unit and two group home units for persons with dementia, and seven nursing home units having a somatic focus. The nursing home has a pronounced focus on person-centred care. For this reason, the older persons living at the nursing home are called “tenants” and not “patients” or “care users”.

The study sought to include an appropriate purposeful sample according to the selection criteria. Inclusion criteria were older persons living alone in a nursing home for at least three months. Participants should have communicative ability, in other words be able to receive and provide information. Exclusion criteria were persons with a diagnosis of dementia (not including mild cognitive impairment which was clinically assessed by nurses) or with such impaired hearing that they were unable to compensate with a hearing aid. First, the older persons were briefly informed about the study by the nurse in charge of their nursing, subsequently a nurse not responsible for the care gave further oral information and the older person also received an informational letter. Thereafter, if the person agreed to participate, they gave their written informed consent. Seventeen persons were invited and ten agreed to participate in interviews. Those who declined to participate did so because of frailty or lack of interest; one person died before the interview.

### Data collection

Nurses working in the nursing home, not responsible for the care of the participants, conducted eight interviews, and due to practical reasons, the researchers conducted two interviews. The nurses had some experience of research but to ensure the quality of data and to ensure consistency of data collection the researcher and the nurses together went through the data collection procedure and the interview guide. The nurses tested the interview questions on their colleagues before they started data collection on the older persons. Each interview was conducted individually in an undisturbed place that the participant themselves chose at the nursing home, usually in their own apartment. A semi-structured interview guide that was based on the research question was used. Example of questions used were: Do you ever feel lonely and are bothered by it? If yes, can you give examples of when you feel lonely? Do you have any suggestions on how to reduce your feelings of loneliness?

The interviews were audiotaped with the participants’ approval and lasted an average of 23 (range 8–43) minutes. The interviews were coded so that they could not be linked to any individual and were subsequently transcribed verbatim by a professional transcriber.

### Data analysis

To captures experiences, a qualitative method was considered appropriate (Patton, 2015). The COREQ checklist facilitated the reporting of the study (Tong et al., 2007). The data analysis was carried out according to systematic text condensation, an inductive analysis based on four phases (Malterud, 2012). In the first phase focused on “overall impressions” (Malterud, 2012), the material was read through individually several times to become familiar with the data. Preliminary themes were first created individually, and after comparison, discussion and consideration, preliminary themes were created communally. These preliminary themes included: The role of the staff, Independence; Barriers to community; and Adaptation.

In the second phase, which Malterud called “Meaning-bearing units—from themes to code groups” (Malterud, 2012, 2015), data material that corresponded to the study aim was identified. The data material was divided into meaning-bearing units and sorted into more than 20 code groups under the preliminary themes identified in the first phase. Examples of code groups were “Being seen”; and “Loneliness doesn’t have to be a problem”. In this phase, preliminary themes were continuously adjusted, to include the different aspects of the same theme. The adjustment resulted in six themes: “The view of loneliness”; “Meaningfulness provides security”; “Challenges in the community”; “Loss”; “Family and friends”; and “Acceptance”. Some themes were renamed or merged to better describe the content of the code groups (see examples in Table I). The code groups were also processed when the content was re-analysed. The coding was done in a spreadsheet on computer.

**Table I.** Example of the analysis process.

Preliminary theme	Meaning-bearing unit	Code group	Adjusted theme	Condensation	Category
**Adaption**	*“I have no problem. I’ve been a lot by myself my whole life, actually, yes not as married, no but as a single person, I’ve been by myself a lot. As long as I have a radio, it’s no problem* [chuckles].” (Interview 5)	“Loneliness doesn’t have to be a problem”	**Acceptance**	“I have no problem being alone and can occupy myself, for example by reading or watching TV. Sometimes it’s difficult to find something I can do; then memories can give me security.”	Acceptance and meaningful existence alleviate loneliness
**The role of the staff**	*“ … and it was so funny when they came then, the staff came and said that > NN wants to talk to you < […] it’s funny that they want to.”* (Interview 2)	”To be seen”	**Meaningfulness provides security**	“The contact with the healthcare staff means a lot to me, they make me feel seen and I feel a sense of purpose in everyday life”	Acceptance and meaningful existence alleviate loneliness

The third phase, referred to by Malterud (2012) as “Condensation—from code groups to meaning”, was carried out by condensing the meaning-bearing units included in the code groups into a common description using the first-person singular pronoun “I” to highlight the content of each individual code group. For example, the meaning within the code group “Loneliness doesn’t have to be a problem” was highlighted by the condensation “I have no problem being alone and can occupy myself, for example by reading or watching TV. Sometimes it’s difficult to find something I can do; then memories can give me security.” During the third phase, some of the code group names were again changed and some were discovered to belong to a different theme. The results were based on ten condensations from ten code groups falling under three themes. A quote from the interviews was selected for each condensation to illustrate the content.

In the fourth and last phase titled “Summary—from condensation to descriptions and concepts” (Malterud, 2012), the condensations were developed into a third-person descriptive text. All meaning-bearing units within each code group were compared with the descriptive text to ensure that no material data content was omitted. The code groups were only a working tool, and the results were described in paragraphs based on previous condensations and presented in three final category headings. Finally, the researchers discussed the final categories until consensus was reached (see Table II).

**Table II.** Final code groups and categories.

Code groups	Categories
“Friends, family and health care professionals reduce loneliness” “Missing loved ones”	Relatives and healthcare professionals’ matter
“Acceptance of life changes” “Being seen” “Meeting in a conversation” “Occupation and community provide meaningfulness” “Loneliness doesn’t have to be a problem”	Acceptance and meaningful existence alleviate loneliness
“Lack of energy” “Loss of functional capacity” “Differences prevent new friendships”	Challenges affecting the experience of loneliness

## Ethical considerations

This study has been approved by the Swedish Ethical Authority (reference number 2020–03878). The study followed the Helsinki Declaration (World Medical Association, 2018) and its principle that caring for the individual must always take precedence over the interests of society and science. Written informed consent was obtained from all participants by the nurse or the researcher conducting the interview, and the older persons were informed that they could withdraw their participation at any time. Before the interview, the participants received a summary description of the project, informing them about the aim of the study. The information was repeated orally before each interview began. As previously mentioned, the interviews were coded to ensure the older people’s anonymity. Person-related data were treated confidentially. Personal data and code lists are available at the University Health Care Research Center at Region Örebro county, which was responsible for the interviews and carried out a risk assessment regarding how to handle the research data. Furthermore, to maintain confidentiality, the data are presented only at a group level.

## Results

The study included two men and eight women aged between 67 and 98 years, who had lived in the nursing home for between 9 months and 6 years. Based on the older persons’ experiences of loneliness, three main categories were identified: “Relatives and health care professionals matter”; “Acceptance and meaningful existence alleviate loneliness”; and “Challenges affecting the experience of loneliness”.

### Relatives and health care professionals matter

All the participants described that family and friends had been and/or still were important in terms of the experience of loneliness. Some had chosen to move to a care home that was geographically close to their loved ones. The participants described having a lot of support from friends and family, who visited and helped with practical things and with whom they did activities outside the care home. They also kept in touch with their relatives by telephone. They felt that the relationships with their loved ones were important because they had known each other for a long time, and they had a lot in common and had shared many experiences before that they could talk about.

The results showed that, for those who did not have loved ones, feelings of loneliness were sometimes reinforced when others talked about events and holidays where they met family and friends. The participants described that they found it difficult to “replace” previous relationships they had lost because the loved ones had stopped contacting them, did not have time to meet, or had died.

My sister died just before Christmas so now I don’t have any of my family left. But you can’t replace her, you can’t. You can’t replace family. (Interview 5)

Further the result showed, the security of having health care professionals nearby. They could come up with things to do and be there in conversations.

There is always someone in the staff . who might bring up something and then you might start with something […] a little bit experimenting with some things then so that you can create some different things with both paper and with . yarn and yes, whatever it is. (Interview 9)

However, older persons also expressed the importance of having known healthcare professionals at the nursing home.

But then people come from other places and it’s not so funny because they don’t know anything here, they can’t even find the sugar cubes (laughs) […] We try to help if we can. (Interview 10)

### Acceptance and meaningful existence alleviate loneliness

Participants who accepted that life had changed and felt that their life had meaning experienced loneliness as more manageable. Moving to a nursing home because of loneliness at home and increased need for care were described by several. However, the participants differed in their feelings and reactions to this situation. Some described that they found it difficult to accept not being able to do things they had done before. They missed doing practical everyday chores and activities and pursuing interests, and this gave them feelings of futility. Some described that they were not comfortable at the nursing home as they lacked a sense of home. A few participants expressed feelings of confinement and said they had found it hard to adapt to the care home’s routines.

We are called residents and I object to that because I’m not – this isn’t my home but … that is, I had to move somewhere … (Interview 2)

Those who felt seen and had a feeling of involvement experienced joy and found it easier to take the initiative to socialize with other people in the nursing home. The presence of a health care professional was perceived to provide security and support. Conversations, both with health professionals and with other residents, were described as important. Often the conversations were about memories. Being able to talk about yourself and hear how others have experienced life provided confirmation to several. It was also described as an activity that did not require much energy.

Yes and then of course it depends a bit on who it is you are talking to. So of course you can sometimes compare a little bit with … For example, if I take any staff, I can compare a little bit with how she has arranged certain things and how I might have arranged certain things when I was about that age and so on. (Interview 9)

Having someone to confide in reduced feelings of loneliness and many participants felt that they could confide in the health care staff. At the same time, they appreciated that there was a difference between staff and other residents and understood that the staff were doing their job by spending time with them. They described themselves as dependent on the health care professionals to create meaningful activities with others. The participants provided specific suggestions for activities such as quiz rounds, read-aloud groups or discussion groups. They highlighted that, to be feasible, the activities needed to be adapted to residents’ interests and functional ability. On the other hand, the majority did not express a need for collective activities, but engaged themselves with reading, watching TV, reflecting on memories, or listening to the radio. They described themselves as individuals who had actively chosen, and valued loneliness or who had become accustomed to being lonely. One of the participants expressed that the interview question of whether they felt lonely was strange, as they had always managed to be on their own quite happily.

### Challenges affecting the experience of loneliness

Various barriers affected the participants’ experiences of loneliness. One of these was poorer energy and desire. In the past, they had met their friends more often, but now they had less desire and energy for social contacts. The need for transportation services and walking aids made going out difficult, and many preferred telephone contact, as even their friends sometimes had difficulties getting out to meet. Having hearing and vision loss were another barrier to community, especially hearing impairment as it made conversations more difficult. The reduced energy also decreased the motivation to create new contacts in the nursing home.

I can’t take it that much, so there’s kind of no point in pulling something big, I do try, but well, no, it’s not something that I feel like I have a huge desire to do now.

(Interview 4)

Several participants had moved to the nursing home in the hope of meeting people and making new friends, but differences in interests and life experiences, age differences or residents’ illnesses stood in the way of development of new friendships. One participant additionally described the fear of getting to know and making friends with someone who might be approaching the end of their life. Although the residents met physically, for example around regular meals, the participants described difficulties in creating social contacts as many residents chose to spend a great part of the time in their individual rooms, making it lonely for those who remained in the communal space.

You sit and eat and drink … All of a sudden, everyone has gone to their rooms. It’s so empty down here […] yes, it’s – sometimes you sit and talk, and stuff like that, but … ehh … I think it’s bad contact, really. (Interview 8)

## Discussion

The key findings were that older individuals experienced different challenges that influenced their experience of loneliness. For example, differences between older persons could prevent friendship. Further, the common opinion among older people living in an academic nursing home were that relatives and healthcare professionals were important for their experiences of loneliness. Moreover, accepting life changes and having access to meaningful activities alleviated the experience of loneliness.

### The importance of meaningful relationships

The results showed that the older persons had difficulties in creating meaningful relationships with the other residents because of different interests and lifestyles, but that relatives, who can often share memories and topics of conversation, became important. The health care professionals were given a compensatory role as persons to talk to in everyday life, although some older persons mentioned that there was a distance between the nursing staff and the residents. According to Schuster (2006), there may be a dilemma between a nurse’s professional approach to their work and empathy with the patient who needs friendship and support. The line between friendship and professional support can become unclear for both parties when it comes to maintaining a good care relationship. Our results showed that the health care professionals were important social contacts for some of the older persons. Care staff therefore need to be aware of the need to listen as a professional way of working that can help alleviate the loneliness of older persons. Emotional closeness to other people is associated with less loneliness, but care workers do not always contribute to alleviating loneliness in older people in nursing homes (Drageset et al., 2011). Lacking meaningful contacts in life is a form of social isolation, and the loss of loved ones is a risk factor for loneliness (Dahlberg et al., 2018; Social Health and Welfare, 2020a). Health care professionals should therefore encourage the contacts that do exist, for example by being available as a support during phone calls, to reduce the risk of social loneliness. The results showed that the older people felt it was important to meet in conversation and that many people’s social contacts had generally decreased over the years. This means that the ability to listen matters a lot. Having someone listening to your meaningful stories reduces loneliness and creates community. Sharing your experiences can bring a sense of hope and healing (Rehling, 2008).

The result showed challenges in terms of loneliness, for example, how differences between the residents underlined the experience of loneliness. The common areas were often empty as most residents retreated to their rooms; some said that they themselves chose to be alone. Sjöberg et al. (2017) describe how older people who do not feel they have anything in common with their new neighbours may paradoxically withdraw to their rooms to reduce the feeling of loneliness. This suggests that being alone, in other words social isolation, can reduce feelings of being lonely as well as existential loneliness. Perhaps this was the main reason why many older people in this study chose the solitude of their rooms over socializing with other residents; they felt existentially alone in a context shared with others they were different from. Eskimez et al. (2019) showed that older people move into nursing homes because of loneliness, but it is unclear whether loneliness is alleviated by this move. Not feeling at home in the nursing home can in fact reinforce existential loneliness (Bolmsjö et al., 2019).

### Managing loneliness can maintain a sense of dignity

The results showed that the older people had different levels of acceptance of their situation. They who experienced meaninglessness seemed to have less drive to actively seek activities or community. Sjöberg et al. (2017) have presented similar results where older people in a nursing home experienced increased feelings of existential loneliness when they lacked a meaningful everyday life or had no one to share life with. The results showed that the older people who had found new everyday tasks and described joy and meaningfulness seemed to have better acceptance of life as it was now. It could be a simple activity such as listening to the radio, as also confirmed by Jansson et al. (2021). Kvaal et al. (2014) likewise have shown that older people deal with loneliness in different ways; some have an inability to change their situation while others like being alone and experience their situation as opportunity for change. Meaningful activities can reduce the negative health effects of loneliness, mainly depression (Lapane et al., 2022). It is therefore important for health professionals to facilitate employment and social interaction, which could help maintain older persons’ dignity. The results showed that the older people experienced loneliness, which, according to Edlund et al. (2013) can undermine a person’s dignity. Absolute dignity includes human dignity, while relative dignity is a variable based on the person’s relationship with values, context and other people. Feeling lonely or no longer being able to do things in the way you have previously done can reduce self-esteem. Choosing loneliness can then be a way to avoid being reminded of the negative feelings that arise from loss of function. Frail older people have described that being trapped in a frail and impaired body can cause feelings of existential loneliness (Sjöberg et al., 2017). When the older person can no longer cope with everyday activities, they need to develop an acceptance of lack of abilities and to learn to see themselves as worthy of the help they require (Edlund et al., 2013). Our results show that the interviewed older persons felt they were able to cope with being alone and some even chose to be on their own, which could indicate that a self-image of being able to fend for oneself protects one’s dignity. Recognizing one’s own values despite changes in functional capacity and accepting this situation shows the importance of feeling relative dignity (Edlund et al., 2013). The results showed that confirmation was important for a more positive experience of loneliness. According to Clancy et al. (2021), the feeling of being seen for who you are and not just in terms of a diagnosis reinforces the dignity of older people. Triggers for existential loneliness are unnecessary waiting, not feeling at home and not being encountered with respect and dignity. Also, seeing and hearing others suffer can give rise to existential loneliness in older people (Larsson et al., 2023). Therefore, the way older people’s loneliness is handled is important in terms of not creating feelings of indignity.

### Living in the memories of a previous life

The results showed that several interview questions about loneliness led to answers about the past and older persons’ memories. The older people longed for their loved ones and their past life, and they cherished their memories. Nostalgia has been described as a kind of longing that contributes to coping to protect against feelings of loneliness and meaninglessness; especially memories of the close family can be soothing (Sedikides & Wildschut, 2019). Longing can be both positive, when it gives hope and faith in the future, and negative, when it focuses on unfulfilled things, when we cannot live our lives the way we want (Delmar, 2006; Jansson et al., 2021). The older persons described a deep loss when talking about relatives who were no longer alive. Such loss could reinforce the experience of not living the life they desired. There were also experiences of confinement and of not feeling at home in the nursing home, which has also been described by Shpakou et al. (2021). A longing for and comparison with the former home can reinforce experiences of loneliness and isolation (Jansson et al., 2021). In older people who only live with a longing for the past, it may be difficult to achieve an acceptance of the present, as they may be creating a negative longing instead of a hopeful aspiration. Health care professionals should confirm an older person in their current existence; this could help the older person to accept their situation and facilitate the alleviation of loneliness.

## Methodological considerations

A qualitative method was considered appropriate to meet the study aim because it is an approach that captures experiences (Patton, 2015). All results that corresponded to the aim were reported regardless of the researchers’ pre-understanding or own opinions. Researchers’ pre-understanding was based on experience of clinical work and ageing research.

The fact that the older persons were asked for participation by a nurse who knew them and was known to them at the nursing home was a strength as it increased the chances of getting an appropriate selection based on the study purpose. However, it may have been a weakness that a familiar nurse asked the older persons for participation, as they may have felt obliged to accept. Yet, the fact that another nurse or researcher then provided additional information and received the consent helped to reduce this risk. The interviews were mainly conducted by a nurse at the nursing home who knew the older persons but worked in another department, which may have reduced the risk of an unfavourable power relation. Nevertheless, it may have affected the familiarity between them and the possibility of a deeper interview.

Further, as the interviews were conducted individually and in privacy gave the older persons a better opportunity to dare to talk about sensitive topics (Malterud, 2012). A limitation was the fact that the study sought to include 15–20 older participants, but only ten finally participated. This can possibly be explained by the fact that many older people suffer from frailty (Warmoth et al., 2016), which makes it more difficult for them to participate in research. According to Malterud et al. (2016), qualitative studies including a low number of participants can still be considered to have informational power. The exclusion of people with severe dementia can be seen as a limitation but was justified with the concern of reliability of interview data and the need for higher functioning while participating in interviews. Cognitively intact participants were considered appropriate to provide insight into loneliness in this study (World Medical Association, 2018).

The interviews were relatively short and sometimes follow-up questions should have been asked but were not; also, in some of the interviews it was unclear whether the older person talked about feeling lonely or about being alone. Possibly longer interviews with the participants would have helped build trust and elicit deeper answers (Malterud, 2015).

Systematic text condensation was considered appropriate as the analysis aimed to highlight diversity and nuances (Malterud, 2015). Systematic text condensation is particularly suitable for interview studies with a limited number of participants, as it explores events and relationships from the participants’ life world (Malterud, 2012). During the analysis process, it was discovered that several categories recurred after only a few interviews, suggesting that the data became sufficiently saturated to achieve reliability and transferability (Malterud, 2012; Polit & Beck, 2021). An important criterion for transferability is that the findings can provide insight into a context other than the one studied (Malterud, 2015). The sample and context may be considered representative of older residents living in nursing homes, which may contribute to the transferability of the results to similar contexts.

Another limitation was that qualitative content analysis in all its forms is based on the interpretation of text, and based on pre-understanding, people can interpret the same text in different ways. Interpretation itself is also an abstract process that is difficult to describe in words (Polit & Beck, 2021). Therefore, throughout the analysis process, a conscious approach was taken regarding the researchers’ pre-understanding concerning previous clinical work, teaching and research within the field, all to increase the reliability of the study (Priebe & Landström, 2017). One weakness of the study was also that the analysis was not validated by an external third party, for example by coding parts of the data material, which could have increased reliability (The Swedish Agency for Medical and Social Evaluation, 2022).

## Clinical implications

District nurses have the responsibility to work with health promotion and adapt care based on the older persons’ individual narratives, while at the same time they need an understanding of the social context and health and protective factors for health. Older people living in nursing homes are different from each other in several aspects, also when it comes to feeling loneliness and how they handle such feelings. Perhaps feelings of loneliness can be reduced if nursing homes are person-centred based on a person’s lifestyle or care needs already at moving in. Based on the study findings, both relatives and health care professionals are important for older persons in terms of experiencing loneliness. Health care professionals need to create a meaningful present existence for the older person to alleviate experiences of loneliness. Health care professionals should also talk and listen in a professional and thoughtful manner and support the older person’s family contacts by enabling telephone calls and physical meetings. The study findings showed that there are challenges affecting older persons’ experiences of loneliness. By creating awareness of these findings’ health care can alleviate older people’s experiences of loneliness through different forms of care which in turn can help reduce both health care costs and the significance of loneliness as a public health problem.

## Future research

A larger study with more and deeper interviews on the subject of loneliness could explore the differences between social and existential loneliness, as several of the older persons answered the question about *feeling lonely* in a way that suggested they thought it was about *being alone*. Further, it would be interesting to carry out a longitudinal study that shows whether loneliness decreases or increases over time after moving into a nursing home. A study aimed at older persons who have no relatives can answer how best to support and alleviate their loneliness.

## Conclusions

Based on our findings, we can conclude that the older persons we interviewed described that they were lonely, but they experienced loneliness in different ways. There was a longing for what had been; however, most also felt that they managed on their own because they had adapted and become accustomed to being, or chose to be, alone, which could be a way to maintain a sense of dignity. Relatives and healthcare professionals played a significant role in the experience of loneliness. Loss of loved ones increased feelings of loneliness in these older persons, even though they lived in a nursing home surrounded by others. Personal differences between the residents often prevented new relationships from being created and instead reinforced the feelings of loneliness. We conclude that meaningful everyday activities counteract negative feelings of loneliness and that care workers have an important function of listening in conversation with the older person to create meaningfulness and affirmation.
